# The evolution of obligate sex: the roles of sexual selection and recombination

**DOI:** 10.1002/ece3.1516

**Published:** 2015-06-04

**Authors:** Maya Kleiman, Lilach Hadany

**Affiliations:** 1 Department of Chemistry, Ben-Gurion University of the NegevBe’er-Sheva, 8410501, Israel; 2 Department of Molecular Biology and Ecology of Plants, Faculty of Life Sciences, Tel Aviv UniversityRamat Aviv, 69978, Israel

**Keywords:** Evolution of sex, facultative sex, obligate sex, sexual selection, simulation

## Abstract

The evolution of sex is one of the greatest mysteries in evolutionary biology. An even greater mystery is the evolution of obligate sex, particularly when competing with facultative sex and not with complete asexuality. Here, we develop a stochastic simulation of an obligate allele invading a facultative population, where males are subject to sexual selection. We identify a range of parameters where sexual selection can contribute to the evolution of obligate sex: Especially when the cost of sex is low, mutation rate is high, and the facultative individuals do not reproduce sexually very often. The advantage of obligate sex becomes larger in the absence of recombination. Surprisingly, obligate sex can take over even when the population has a lower mean fitness as a result. We show that this is due to the high success of obligate males that can compensate the cost of sex.

## Introduction

The evolution of sex is one of the major open questions in evolutionary biology. Sexual reproduction carries high costs: First, all else being equal, an asexual population has a twofold advantage in fitness over a sexual population in which males contribute nothing but their genomes to the next generation – the “twofold cost of males” (Maynard Smith 1971; Michod and Levin 1988). Second, finding mates requires time and energy. Finally, recombination may break down existing genetic associations generated by natural selection, leading to “recombination load” (Barton and Charlesworth 1998; Otto and Lenormand 2002; Roze and Michod 2010). Yet most higher eukaryotes engage in sexual reproduction, at least part of the time.

Many models explaining the advantage of sexual reproduction over asexuality have been proposed, including the purging of deleterious mutations (Fisher 1930; Kondrashov 1988), adaptation (Muller 1932; Bell 1982; Peck 1994; Otto and Barton 1997; Waxman and Peck 1999; Hadany and Feldman 2005; Becks and Agrawal 2010; Hartfield et al. 2010), host–parasite co-evolution (Hamilton et al. 1990; Howard and Lively 1994), random genetic drift (Muller 1964; Otto and Barton 2001; Iles et al. 2003; Barton and Otto 2005; Keightley and Otto 2006; Roze and Barton 2006), and pluralistic models combining some or all of the above (West et al. 1999).

Most of these models found that there was an advantage for a low frequency of sexual reproduction over complete asexuality. Very few models exist explaining the evolution and maintenance of obligate sexuality in comparison with facultative sex (Peck and Waxman 2000) (and see (Hörandl 2009) for review) including spatial (Lenormand and Otto 2000) and temporal (Halkett et al. 2006) models. In fact, according to most models, organisms that engage in sexual reproduction only part of the time seem to have the best of both worlds (Charlesworth et al. 1993; Green and Noakes 1995; Hurst and Peck 1996; Corley et al. 2001; Hadany and Beker 2003a,b; Yamauchi and Kamite 2003; Hadany and Otto 2009; D’Souza and Michiels 2010)).

One likely candidate for influencing the evolution of obligate sex is sexual selection, the process by which individuals of one sex, typically the males, compete over mating opportunities (Darwin 1906). This process offers a direct advantage to fit individuals – more offspring for males, and more grand-offspring (and later generation progeny) for females through the reproductive advantage of their sons (Hadany and Beker 2007). Sexual selection can take the form of males competing over females or territory (Andersson 1994) or females selecting the most attractive male, for example, the one with the most impressive tail or the longest neck (Cronin 1993; Senter 2007). If individuals pick the best mates they can find rather than mating randomly (Huxley 1863; Dall et al. 2006) then sexual populations can gain an advantage over asexual ones (Agrawal 2001; Siller 2001).

The advantage of obligate sex is hardest to explain when there is gene flow between the facultative and obligate populations. Intuitively, it seems that the facultative population has all of the above-mentioned advantages, and in addition, it is also capable of gaining good genes from interbreeding with the obligately sexual population. In this situation, the obligate population pays the full cost for sex, yet passes on some of the benefits of sex to the facultative population. Recently, it has been shown that sexual selection allows an allele for increased sexual reproduction to invade a facultative population as long as sexual selection and natural selection are acting in the same direction (Roze and Otto 2012). Although this work did not show a twofold advantage for the obligate population, probably because natural selection was too weak. Strong sexual selection can even explain the advantage of obligate sex invading a facultative population (Hadany and Beker 2007), although only for a haploid population with free recombination. None of the previous works studied the evolution of obligate sex together with its effect on mean fitness.

In this article, we develop stochastic simulations, extending the deterministic model of Hadany and Beker (2007). We consider the evolution of obligate sex in a finite diploid population, with varying levels of recombination and dominance, and examine its implications for the whole population.

## Methods

We used individual-based simulations to study the dynamics of obligately sexual mutants invading a facultatively sexual population. The population is made up of N diploids, each possessing two copies of k chromosomes. Each chromosome is represented by a set of integers indicating the positions of deleterious mutations present on the chromosome. The whole genome consists of 10,000 biallelic loci, in order to mimic a higher eukaryotic genome. The viability (*v*) of an individual is determined by:

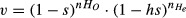
1where 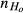 and 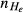 are the numbers of deleterious mutations present in homozygous and heterozygous states, respectively. The genome further includes a modifier locus with two alleles: F (for facultative sex) and O (for obligate sex). The location of the modifier within the genome is chosen at random for each run. Mutation at the modifier locus is assumed to be rare during the invasion process.

An OO individual reproduces sexually at all times with a 50% chance of being either a male or a female. A FF individual has a probability *P*_asex_ (FF) = *a* of reproducing asexually as a female and a probability *P*_sex_ (FF) = 1 − *a* of reproducing sexually with a 50% chance of being either a male or a female. An OF individual has a probability *P*_asex_ (OF) = (*a*/2) of reproducing asexually and a probability *P*_sex_ (OF) = 1 − (*a*/2) of reproducing sexually. The reproductive mode and gender of each individual are determined once in its lifetime, shortly after birth.

The next generation is produced in the following way: For each of the *N* individuals of the next generation, it is first determined whether the new individual was generated through sexual or asexual reproduction. The probability that the offspring results from a sexual reproduction event is as follows:

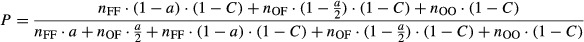
2where *n*_*ij*_ is the number of individuals with genotype *ij* at the modifier locus and *C* is the cost of sex.

A mother with the appropriate reproductive mode is then chosen randomly. That is, for an offspring resulting from asexual reproduction, a mother is chosen from the asexually reproducing individuals and produces a copy of her genotype. For an offspring resulting from sexual reproduction, a mother is chosen from the sexually reproducing females and a father is then chosen from the males. The chances of any given male being chosen by a sexually reproducing female is proportional to its sexual fitness, *s*_*i*_, where

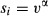
3and α modulates the strength of sexual selection on the males relative to natural selection (α > 1 implying sexual selection is stronger). Each of the sexual parents then forms a gamete through segregation and recombination. In the presence of recombination, a single cross-over occurs at a random location in each chromosome, so the recombination rate is determined by the number of chromosomes. We also tested the case of one chromosome with no recombination. The gametes of the two parents then combine to form an offspring. The offspring (produced either sexually or asexually) then undergoes mutation. The number of new mutations is sampled from a Poisson distribution with parameter U, and the position of each mutation is sampled from a uniform distribution. The viability – the chance of the offspring surviving to maturity – is then calculated as stated above (Eq. 1), as is the sexual fitness (Eq. 3). Sexual selection acts only if the offspring matured into a male and can be selected by a female. The next generation is considered to be formed as soon as N new individuals survive.

We ran two types of simulations, one to explore the evolution of the rate of sex and the second to explore the fitness consequences of a particular frequency of sex. In the first type of simulation, we started with a pure facultative (FF) population with probability *a* for asexual reproduction, which was allowed to reproduce until mutation-selection balance was achieved. At this point, 5% of the individuals, chosen at random, switched their reproductive mode and became obligately sexual (OO). The process was continued until one of the following three outcomes was observed:

1. The obligate allele (O) took over (more than 99.9% of the population).
2. The obligate allele (O) disappeared (less than 0.01% of the population).
3. 10,000 generations had passed and both alleles were still present within the population. When that result was obtained, it was confirmed by rerunning this set of parameters once for 100,000 generations.

The mutation rate (*U*), the cost of sex (*C*), and the strength of sexual selection (α) were held constant throughout the run. Similarly, we considered the reverse invasion, simulating an OO population until it reached steady state, at which point 5% of the population was switched to FF. Three runs in each direction were performed. If the three runs did not yield the same result, four additional runs were performed in each direction. Polymorphism was chosen as the outcome only when both types of invasion resulted in polymorphism in at least three runs in each direction. All results were statistically significant (*P* < 0.05) except a few sets of parameters, all with *C* = 0, that did not yield statistically significant results, even after ten runs in each direction. Those sets of parameters are noted in gray in the relevant figures.

In the second type of simulation, two separate populations were considered for each parameter set: First, a pure facultative population reproduced until mutation-selection balance was achieved, and then the mean fitness of the population 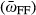 was calculated as:

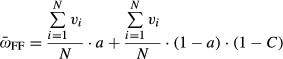
4where *v*_*i*_ is the viability of the *i*’th individual in the population.

Second, an obligately sexual population reproduced under the same conditions until it reached mutation-selection balance, and then the mean fitness of the population 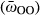 was calculated as:

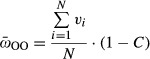
5

These runs were repeated 50 times, and the fitter population was determined using a *t*-test with two-tailed *P* < 0.05. Only one set of parameters did not give a statistically significant result even after 50 runs (noted in orange). In our simulations, *N* = 6000 unless otherwise noted. Our simulation program was written in C++ and is available as supplementary materials using Dryad at http://dx.doi.org/10.5061/dryad.7p649.

## Results

**Figure 1:**
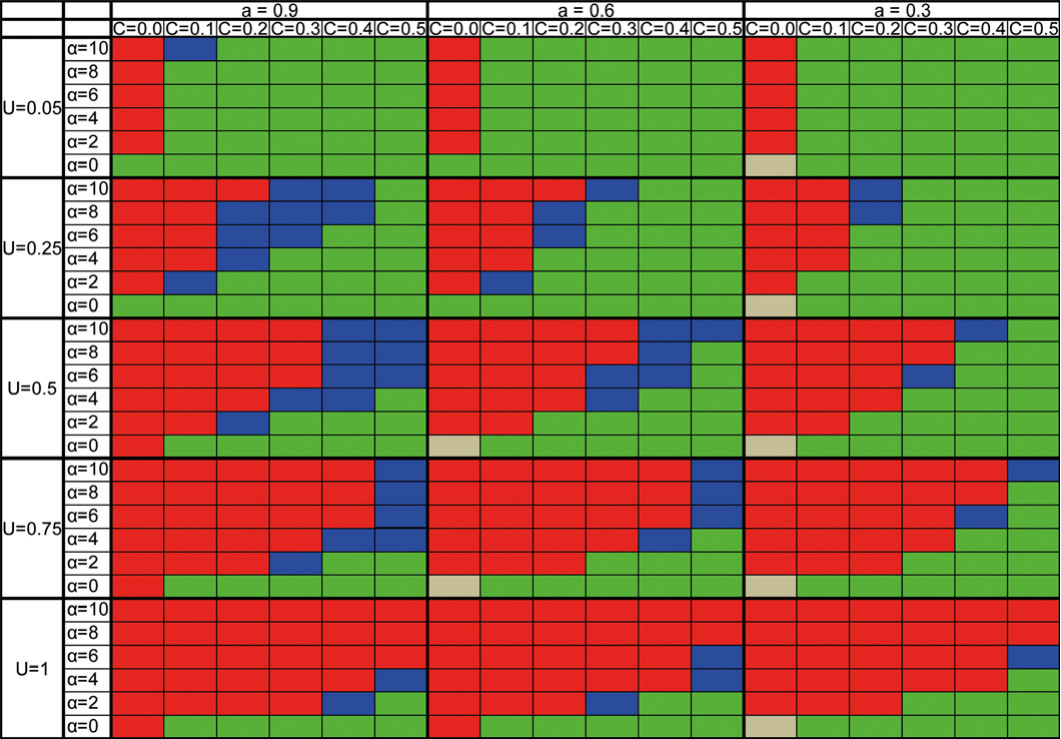
The parameter range for invasion of the obligate modifier. The range of parameters for which the allele for obligate sex takes over (red), disappears (green), or polymorphism is maintained (blue) when invading a facultative population. Each square represents the winning strategy in this set of parameters (see Methods for details). Sets of parameters that did not result in a statistically significant winning strategy are marked in gray. U is the mutation rate, *a* is the probability that a FF individual reproduces asexually, α is the strength of sexual selection, *C* is the cost of sex. *N* = 6000, and the parameters of natural selection are *s *=* *0.2 and *hs* = 0.1.

We found that obligate sex can invade a facultative population, even when sex is associated with substantial costs (Fig.1). Such an invasion is more likely to occur when the mutation rate is high, the cost of sex is low, sexual selection is strong, and *a*, the probability for asexual reproduction among the facultatives, is high. In the absence of sexual selection, obligate sex can evolve only if the mutation rate is very high and the cost is very low, which is consistent with classical models (Kondrashov 1984). Sexual selection can increase the advantage of obligate sex significantly due to more effective elimination of deleterious mutations and also by granting a greater advantage to high-quality males (more often the obligate males). The impact of *a* can be understood in two ways: The larger *a* is, the less sexual the facultative population is and the case becomes more similar to the classical case of sex versus complete asexuality. The other way to view this is the fact that with a large *a*, there is less mixture between the facultative and obligate populations; hence, the facultative population is less likely to enjoy the benefits of advantageous alleles derived from the obligate population. We also found that there is a range of parameters for which the obligate sex allele is maintained at a stable polymorphism (Fig.1). The maintenance of polymorphism is further investigated later on (see Fig. 3). Additional parameters affecting the takeover of the obligate allele, such as recombination, dominance of deleterious alleles and natural selection, are investigated later (Figs. 6).

**Figure 2:**
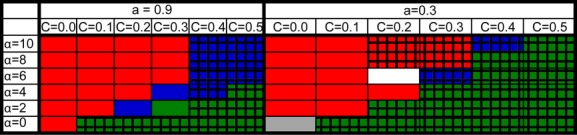
Mean fitness differences and direct competition results between obligate and facultative sex. Comparing the differences in mean fitness (cost of sex is included) between an obligately sexual population and a facultatively sexual population and the ability of the O allele to take over a mixed population. An invasion can accompany an advantage in mean fitness (red) or could occur despite a reduction in mean fitness (dashed red). Elimination of the obligate sex allele can happen in conditions where the obligate population is less fit (green), but also when it is fitter (dashed green). Polymorphism may occur when the obligate population has a higher (blue) or lower (dashed blue) mean fitness. For one set of parameters, the winning strategy could not be determined because the mean fitness of the two populations was not statistically significant (white). For each set of parameters, fifty different runs of pure obligate and pure facultative populations were performed, and the fitter population was determined (see Methods section). Results for the takeover of the O allele were taken from the runs described in Fig.1. The set of parameters for which a winning strategy could not be determined is marked in gray. All runs were performed with mutation rate (*U*) = 0.5 and *N* = 6000. *a* is the probability of a FF individual reproducing asexually, α is the strength of sexual selection, and C is the cost of sex. The parameters for natural selection are *s *=* *0.2 and *hs* = 0.1.

We compared the mean fitness of an obligately sexual population to that of a facultatively sexual population under various parameters in order to test whether the short-term success in taking over the population is accompanied by an average fitness advantage when separated (Fig.2). The mean fitness of the two distinct populations was measured as described in the methods. The individuals in the obligately sexual population carry better genes than the ones in the facultative population at any set of parameters, however, as there is a cost to be paid for sex, this advantage at the genetic level does not always come to light at the overall fitness level. The mean fitness of the pure obligate population significantly exceeds that of the pure facultative population when the cost is low, and the opposite is true when the cost is too high. The threshold cost increases with *a*.

**Figure 3:**
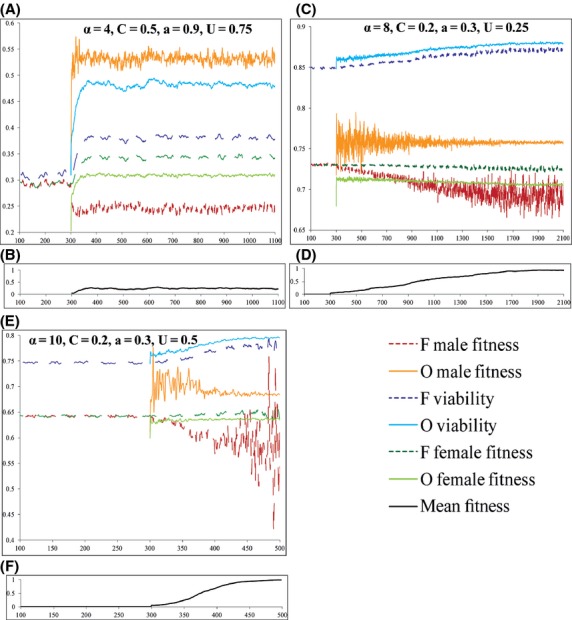
Fitness components of different alleles and genders over time in polymorphism conditions. Two typical runs for polymorphism conditions and one of O taking over despite its long-term disadvantage are presented where 5% OO individuals were introduced to an FF population at steady state. Shown are the viability of juveniles carrying F (dashed blue) and O (light blue) alleles, female fitness of F (dashed green) and O (light green) alleles and male fitness of F (dashed red) and O (pink) in three conditions: high *a*, cost, and mutation rate with low sexual selection (parameters listed on the top of the graph) (A); low *a*, cost, and mutation rate with high sexual selection (α) (C); and one set of parameters where O allele takes over despite the lower mean fitness of the OO population in comparison with the FF population, with low *a*, low cost, and very high sexual selection (E). The percentage of the O allele over time is also shown (B, D, and F). While the O allele becomes associated with better genes, the fitness of O females is lower due to the cost of sex. This is compensated for by the higher success of O males, resulting in a polymorphic state (A–D) or even the takeover of O allele (E–F). The parameters for natural selection are s = 0.2 and hs = 0.1.

We found that the parameter range allowing the invasion of obligate sex is not identical to the parameter range for which the mean fitness of the obligate population is higher. First, there were cases where the mean fitness of the obligate population exceeded that of the facultative population, yet the obligate allele disappeared from the mixed population (Fig.2 dashed green). This can be explained by the fact that the individuals who “pay” less for reproduction (facultatives) can still benefit from the good genes of the obligate individuals, through occasional sexual reproduction and recombination with them. This would make the takeover process of the obligate allele more difficult, especially for relatively low α. Interestingly, there were also cases for which the mean fitness of the obligate population was lower, yet the obligate allele managed to take over the population when competing with a facultative allele (Fig.2 dashed red). The reason for this probably lies with the advantage of obligate males when sexual selection is very high (Fig.3). In such cases, a small advantage in genetic quality translates to a significant advantage in male reproductive success. As a result, the obligate males might father a disproportionate fraction of the offspring. It should be noted that the differences in mean fitness between the two populations were less than 5% in all these cases, suggesting that O takeover does not usually occur with a severe long-term disadvantage. This is demonstrated in Fig.3E.

To further explore the different forces involved in the evolution of obligate sex, we tracked the dynamics of several nonintuitive cases. Figure3 shows typical runs of two cases where stable polymorphism was maintained between facultative and obligate sex, and one case where obligate sex took over while reducing the average fitness of the population. We tracked a number of parameters over time. First, we monitored the mean viability of the F allele 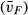 and the mean viability of the O allele 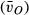, a measure of the quality of the genome associated with the modifier, separately:

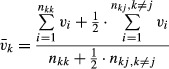
6where *n*_*kj*_ is the number of individuals with genotype *kj* at the modifier locus, and {*k*, *j*} ∈ {*F, O*}.

In order to take into account the cost of sex, we calculated mean female fitness (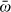
♀_k_) associated with each allele (*k*):

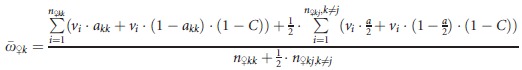
7where *v*_*i*_ is the viability of organism *i*, *n*_♀*kj*_ is the number of females with genotype *kj* at the modifier locus, and *a*_*kk*_ is the probability of a *kk* individual reproducing asexually. The probability of a heterozygous individual reproducing asexually is always *a*\2.

To calculate average male fitness associated with each allele 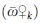, we also took into account the probability of a male being selected for reproduction:

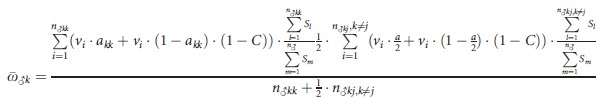
8where *s*_*i*_ is the sexual fitness of male *i*. Finally, we tracked the percentage of the O allele over time within the population.

In all three cases shown in Fig.3, we found that the O allele became associated with better genes (resulting in higher viability). The introduction of O also increased the effectiveness of selection in the F subpopulation, but not to the same extent. In contrast, the fitness of the F females exceeded that of the O females under these parameters due to the reduced cost of sex (Fig.3A, C, and E). However, O males were more likely to be chosen as mates and therefore had a higher fitness than F males (Fig.3A, C, and E). The differences between the F and O alleles are strongly influenced by *a*, as the lower *a* is, the more similar and more mixed O and F populations are (compare Fig.3A and C). A component in this equilibrium may lie in the heterozygote OF individuals. Whenever one of the alleles has a low frequency in the population, it will mostly be found in a heterozygous state with intermediate phenotype. When the optimum is closer to the heterozygote phenotype, negative frequency-dependent selection would result. The range of polymorphism is thus wider when the difference between the facultatives and the obligates is larger, or when *a* is higher (see Fig.1). We find that stable polymorphism may be sustained for a long time. For the sets of parameters that resulted in polymorphism, we ran the populations for 100,000 generations. The polymorphism was maintained in all cases (data not shown). Nevertheless, we would expect that eventually a facultative mutant with a lower *a* might be able to invade the population. As the O percentage increased, a higher fraction of F alleles was found in a heterozygous state (right hand side of Fig.3C and E) resulting in a slight increase in viability and a parallel decrease in female fitness. Similar patterns were observed in multiple runs with the same parameters.

**Figure 4:**
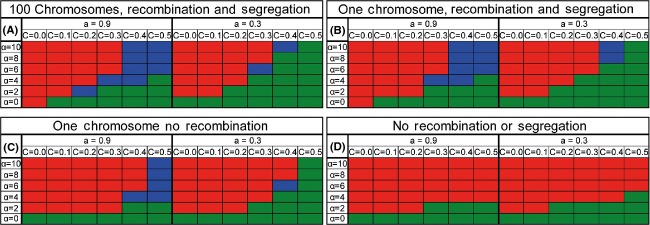
The effect of recombination, segregation, and interbreeding on the ability of the O allele to take over. The range of parameters in which the O allele takes over a facultative population is presented. Red – O takes over, green – O vanishes, blue – polymorphism. (A) Recombination and segregation of 100 chromosomes (100 cross-over events). (B) Recombination and segregation of one chromosome. (C) Segregation of one chromosome with no recombination. (D) No segregation and no recombination (i.e., either the maternal or paternal genome is replicated, with equal probability). When the populations are interbreeding, the higher recombination rate makes it more difficult for the O allele to take over. Parameters: *U* = 0.5, *N* = 6000, *s* = 0.2, and hs = 0.1.

We next wanted to examine the effects of recombination and segregation on the ability of the O allele to take over the population. We first changed the rate of recombination by changing the number of chromosomes from 100 to 1 (Fig.4A and B). We also investigated the case where sexual reproduction involves segregation of one chromosome but no recombination (Fig.4C). An additional control of no segregation and no recombination was considered (Fig.4D), in which a sexual individual finds a mate using sexual selection, but then the individual reproduces asexually (where the asexual offspring has a 50% chance of being formed by the female and a 50% chance of being formed by the male). We found that the lower the recombination rate is, the easier it is for the O allele to take over. Recombination may interfere with the invasion of the O allele because it increases the effectiveness of natural selection in the facultative population (Rice and Chippindale 2001). In addition, recombination also transfers good genes from the obligate to the facultative population making the invasion of the O allele harder. When the populations do not benefit from either recombination or segregation (and are also completely separated), no stable polymorphism occurs, and the fate of the allele is mostly dependent upon α, the sexual selection parameter (Fig.4D). The higher α is, the easier it is for the O allele to take over in that scenario. This is due to the fact that in this case the only benefit of sex derives from sexual selection. Interestingly, when the sexual strategy locus was not linked to the genome, but rather was freely recombining, the ability of the O allele to take over did not depend upon the recombination rate. We tested 1, 20 or 100 chromosomes, with the strategy allele being a separated chromosome, and all gave results identical to the results of the 100 chromosomes where the location of the strategy allele is chosen at random shown in Fig.4A (results not shown). This suggests that the main effect of recombination in this system is the recombination between the modifier locus and the good (or bad) genes linked to it.

**Figure 5:**
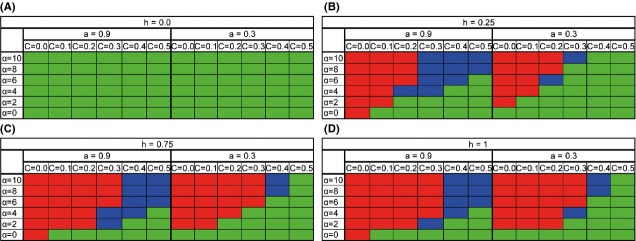
The effect of dominance on the invasion of the obligate modifier. The range of parameters in which the O allele takes over a facultative population is presented. Red – O takes over, green – O vanishes, blue – polymorphism. (A) Deleterious mutations are completely recessive – h = 0. (B) Partially recessive – h = 0.25. (C) Partially dominant – h = 0.75. (D) Completely dominant – h = 1. For all panels: *U* = 0.5, *N* = 6000, *s *=* *0.2.

In order to examine the effect of dominance on our system, we ran the same simulation, varying the dominance of the deleterious allele (Fig.5). We changed h to the two extremes: 0 – representing recessive deleterious mutations (Fig.5A) and 1 – representing complete dominance of the deleterious mutations (Fig.5B). We also looked at intermediate situations: h = 0.25 and h = 0.75 (Fig.5B and C). It seems that the more dominant the deleterious mutations are, the easier it is for an obligate allele to invade the population. This phenomena come to extreme when h = 0, in which case the obligate allele cannot invade at any cost or sexual selection parameter. At h = 0, there is an accumulation of deleterious mutations at mutation-selection balance. The associations between the deleterious mutations can be broken by sex, which has been shown to have a large effect on the ability of sex and recombination to takeover a population, as was shown by Roze (2014). However, in this simulation, it appears that a small amount of sex (10%) is sufficient to gain these advantages and obligate sex is not necessary even with no cost. Sexual selection does not assist in the takeover of the obligate allele as this higher selection has no effect when the fitness is only influenced by two copies of the deleterious allele. These results correspond to Roze and Michod (2010), where in the absence of sexual selection sex is selected against if h < 0.5. While in the presence of sexual selection the effect of dominance is relatively small, it appears that drift comes into play in the case of h = 0 and abolishes any effect of the sexual selection.

**Figure 6:**
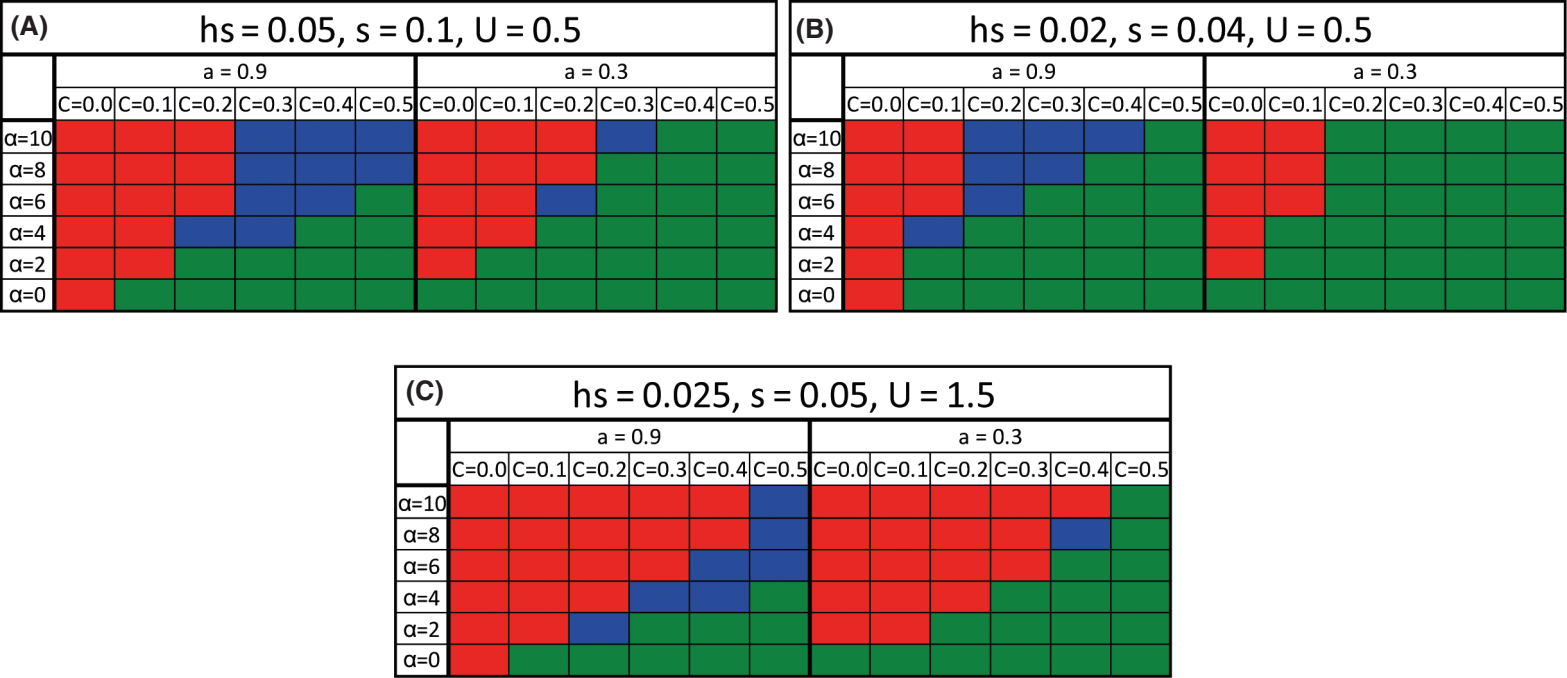
The effect of natural selection on the ability of the O allele to take over. The range of parameters in which the O allele takes over a facultative population is presented. Red – O takes over, green – O vanishes, blue – polymorphism. Parameters for natural selection and mutation are listed on top of each panel. We see that under weaker selection the takeover of the O allele was more difficult (compare A and B) and needed higher sexual selection and lower cost.

In order to be able to compare our results with the results of Roze and Otto (2012), we lowered the strength of natural selection. As can be seen in Fig.6, the parameters for natural selection have a large influence on the ability of the O allele to take over. Decreasing natural selection, while keeping mutation rate the same, resulted in a significantly narrower range of parameters in which obligate sex can take over (compare Fig.6A and B). However, keeping natural selection low while increasing mutation rate, widens the range of parameters in which the obligate allele can take over (compare Fig.6B and C). The lower natural selection is, the lower the classical advantages sex has over complete asexuality. Hence, obligate sex struggles harder to take over and more extreme conditions are needed. The conditions necessary for takeover of the O allele at low natural selection are high mutation rate, high sexual selection, and low cost (Fig.6). Here, we used the same parameters for natural selection as Roze and Otto (2012). It is only possible to compare our results directly to Roze and Otto when *α* = 0. Under these conditions, with no cost for sex, our results were in complete agreement with theirs: We found that obligate sex took over the population when the initial rate of sex is 0.1, but not when the initial rate of sex is 0.7, and they found the equilibrium rate of sex to be 0.8. The results imply that the takeover process of obligate sex depends on the difference between the initial and the equilibrium rates of sex. As cost increases, the equilibrium rate of sex decreases and with cost = 0.1 Roze and Otto found an equilibrium rate of sex of 0.3, and obligate sex was unable to take over the population in our model. In agreement with their results, we found that increasing the selection for males only will increase the probability for obligate sex to take over (just as it would increase the equilibrium rate of sex).

A slightly different algorithm for choosing males was carried out as well, in which each male’s sexual fitness was evaluated only once. If the male’s sexual fitness (given by Eq. 3) was higher than a uniformly drawn number between 0 and 1, he was allowed to reproduce; otherwise, he remained in the population but produced no offspring. The two algorithms gave identical results. Furthermore, a reduction of the population size to 1000 had no significant effect on the results.

## Discussion

In this paper, we demonstrate that obligate sex can evolve in finite diploid populations in the presence of sexual selection, with or without a long-term advantage. We find a parameter range allowing the invasion of a rare obligate allele into a facultative population. The invasion is more likely to occur when mutation rate is high, the cost of sex is low, sexual selection is strong, and the facultative individuals rarely reproduce sexually. Sexual selection is critical to the results: In its absence, even a low cost of sex is enough to hinder the evolution of obligate sex. The rate of recombination significantly influences the ability of an O allele to take over as well. The lower the recombination rate (between the genes determining the viability of the individual and the modifier determining reproduction strategy), the easier it is for an O allele to take over when facultative and obligate individuals are allowed to interbreed. Finally, we find that the short-term advantage of obligate sex, enabling it to take over, is not always accompanied by a long-term advantage. There are sets of parameters for which the obligate population is less fit than the facultative one (due to the cost of sex), yet the O allele takes over, as the obligate males, that tend to carry better genes, are more likely to be chosen as mates by both obligate and facultative females.

Here, we consider the case that natural and sexual selection act in the same direction (“good genes” model). As an obligate allele is introduced into a facultative population, its carriers experience stronger selection, resulting in better sets of genes. Due to competition and gene flow, selection intensifies for the facultative subpopulation as well, but less dramatically. However, the facultative females pay a lower cost for reproduction and hence can have a higher fitness than obligate females. Obligate males, in contrast, have a higher fitness than facultative males, and sexual selection amplifies small advantages in viability into large advantages in the probability of being selected by a female for reproduction. Obligate males thus father a disproportionately high fraction of the sexual offspring in mixed populations. As the percentage of obligates within the population increases, the viability increases, but the fitness of the entire population may increase or decrease (due to the high cost of sex).

Several previous works considered the role of sexual selection in the advantage of sex. In 2001, Agrawal and Siller showed that sexual selection can explain the population level advantage of sexual reproduction by looking at the mutation rate and sexual selection which enable a sexual population to have a higher mean fitness than an asexual population at equilibrium, compensating for the twofold cost of sex. In 2006, Salathé found that sexual populations are immune to complete asexual mutants only under strong sexual selection or high mutation rate, which is consistent with our results. Recently, a few works studied the conditions for an obligate allele to invade a facultative population in the face of significant costs, mostly with analytical models (Hadany and Beker 2007; Roze and Otto 2012).

Here, we study for the first time the interaction between the short-term selection acting on an allele for obligate sex and its long-term effect on the mean fitness of the population. We find that short-term and long-term selection often disagree, in both directions: As expected, we find that obligate sex can be eliminated from the population despite a long-term advantage when the populations are allowed to interbreed. Surprisingly, we also find that obligate sex might sometimes evolve despite a long-term disadvantage. Figure2 shows that there are conditions in which the obligate allele will take over, despite the fact that the average fitness of an obligate population in steady state is lower than that of a facultative population. This counterintuitive result suggests that sex may, after all, have a “selfish” component. The case usually considered hardest for explaining the evolution of obligate sex – the case where the obligate allele is in contact with a facultative population that allegedly makes the best of both worlds – was shown to allow a unique selfish advantage for obligate sex in cases of strong sexual selection.

Our invasion results are qualitatively similar to the results obtained by the analytical model (Hadany and Beker 2007); in the analytical model, an infinite facultative haploid population undergoing free recombination was invaded by an obligate allele. The conditions for takeover of the obligate allele were harsher (lower cost and *a*, higher mutation rate and sexual selection) than the ones shown here. These differences can be put down to our use of finite populations, allowing drift, including Muller’s ratchet (Muller 1932, 1964) and genetic hitchhiking (Barton 2000), factors known to enhance the advantages of sexual reproduction over complete asexuality. In addition, we used realistic recombination rates here, in contrast to free recombination in the analytical model. Finally, we used strong natural selection in most of our simulations, to limit computation time.

The model we present here differs from (Hadany and Beker 2007) in two significant ways: First, we study different rates of recombination here and distinguish the effect of recombination and segregation from that of the sexual selection. We show that even in the absence of recombination or segregation, strong sexual selection can aid the invasion of an obligate allele into a population. In fact, recombination only damages these attempts. Recombination, however, is mostly relevant between the modifier locus and the rest of the genome, consistent with the recent analysis by Roze (2014). The recombination rate determines the quality of the genome. The sexual rates of the modifier determine the escape chances from a bad genomic background. And indeed, here we show that it is the recombination between the modifier determining sexual rates and its genetic background that determines the probability of obligate sex taking over the population.

The second way in which our model differs from that of (Hadany and Beker 2007) is that we modeled a diploid population. It seems that diploidy, in particular the dominance of deleterious mutations, plays a role in determining the conditions under which an obligate allele will be able to invade a facultative population. The more recessive a deleterious mutation is, the harder it is for an obligate allele to invade a facultative population. This is in full agreement with (Roze and Michod 2010) who showed that drift in a finite population favors sex when the deleterious mutations are dominant or partially dominant, but disfavors sex when deleterious mutations are recessive (h < 1/2).

Roze and Otto (2012) found conditions under which obligate sex invaded a facultative population when selection went in the same direction for both males and females, but not with a twofold cost. Their study differs from ours in several ways: We considered a wider range of sexual selection values, up to *α* = 10; stronger natural selection; a smaller population (usually 6000); and rare mutations at the modifier locus (our modifier locus does not mutate, while theirs does). Our results suggest that only with strong natural selection will obligate sex establish with a twofold cost (see Fig.6). Additionally, the obligate allele takes over at very high sexual selection values, some of which were not investigated by Roze and Otto. We found a set of parameters for which a stable polymorphism is maintained between obligate and facultative sexuality. It can be further investigated whether such a polymorphism does exist in real populations under those particular conditions.

In this work, we obtained relatively strong results (evolution of obligate sex in the presence of the twofold cost of sex and interbreeding with facultatives) under strong selection, both natural and sexual. The high parameter values required for the obligate allele to take over a facultative population suggest that although sexual selection may play an important role in the evolution of obligate sex, it is most likely not the only player. Other conditions, such as epistasis (Kondrashov 1988), adaptation in changing environments (Waxman and Peck 1999), and co-evolution with parasites (Howard and Lively 1994), are probably important as well. High mutation rates may also have a large effect, especially as recent studies have shown that the expected mutation rate in mammals might be as high as 2.2 for a diploid genome per generation in humans ((Keightley 2012) and see (Kondrashov and Kondrashov 2010) for review). A pluralistic theory (West et al. 1999) taking into account sexual selection and other conditions is needed for a full resolution of obligate sex evolution.

## Supporting Information

**Data S1**. simulation code.
